# Elastic intramedullary nailing for recurrent radial fracture associated with persistent ulnar bowing deformity in a pediatric patient: a case report

**DOI:** 10.3389/fped.2026.1911085

**Published:** 2026-08-26

**Authors:** Wenrong Yang, Guangmin Pu, Zhigang Zhao, Shuchun Yang, Zhijun Chen, Jincai Liu, Dan Wang

**Affiliations:** Department of Orthopaedics, Chuxiong Yi Autonomous Prefecture Hospital of Traditional Chinese Medicine, Chuxiong, China

**Keywords:** case report, elastic intramedullary nailing, pediatric patient, recurrent radial fracture, ulnar bowing deformity

## Abstract

**Background:**

Pediatric forearm bowing fracture is an occult incomplete injury with a high misdiagnosis rate. Persistent ulnar bowing deformity is a potential risk factor for recurrent radial fracture due to altered forearm biomechanical stress distribution, and unified management protocols for this combined injury remain lacking. This case report describes the diagnosis and treatment of an 8-year-old girl who sustained a second ipsilateral radial fracture four years after an initial forearm trauma accompanied by ulnar bowing deformity at 4 years old, aiming to provide practical clinical evidence and generate preliminary treatment hypotheses for this clinical scenario.

**Case presentation:**

The patient, an 8-year-old girl, first presented at 4 years old with a middle-distal third radial fracture concurrent with ulnar diaphyseal bowing deformity. Initial management consisted of closed manual reduction and splint immobilization, and no corrective intervention was performed for the ulnar bowing deformity. Four years following the initial injury, she fell and sustained a displaced recurrent radial fracture at the middle-proximal third of the same forearm. Closed manipulative reduction and plaster casting failed to sustain adequate fracture alignment, prompting surgical intervention with open reduction and elastic intramedullary nailing to repair the radial fracture; the pre-existing ulnar bowing deformity was left unaddressed intraoperatively. At the two-month postoperative follow-up, elbow and forearm function were fully preserved, with only mild residual forearm angular deformity and no functional deficits noted. No surgical complications occurred, and the family tolerated the mild residual deformity.

**Conclusions:**

This single case observation generates a preliminary clinical hypothesis rather than universal clinical guidance: Elastic intramedullary nailing serves as a safe and reliable minimally invasive surgical option for children with recurrent displaced radial fractures when conservative treatment yields unsatisfactory reduction maintenance. Additional corrective osteotomy is not indicated for pediatric patients presenting with mild ulnar bowing deformity, normal inner-epiphyseal ulna-radius length ratio. Targeted treatment focused solely on the radial fracture can achieve satisfactory radiological recovery and short-term functional outcomes in this patient population.

## 1 Introduction

Pediatric forearm bowing fractures are a unique type of incomplete orthopedic injury caused by the high organic content and good plasticity of pediatric bone, which results in plastic bending rather than complete fracture under low-energy trauma (1). These fractures lack obvious fracture lines, leading to a high clinical misdiagnosis rate, and even mild uncorrected ulnar bowing deformity can alter forearm interosseous membrane tension and stress distribution (2, 3). While radial bowing deformity is well recognized to impair forearm rotation function, the association between uncorrected ulnar bowing deformity and recurrent radial fracture is poorly documented in the literature, and no clear clinical treatment guidelines exist for this combined injury (4, 5). This case reports an 8-year-old child who developed a recurrent radial fracture four years after sustaining an initial combined radial fracture and ulnar plastic bowing deformity at 4 years old(uncorrected ulnar deformity), and was successfully treated with open reduction and elastic intramedullary nailing for the recurrent radial fracture (without ulnar deformity correction). This long-term follow-up case bears unique clinical value. It documents recurrent radial fractures secondary to untreated ulnar bowing deformity and verifies the efficacy of minimally invasive elastic intramedullary nailing for such injuries. Meanwhile, it puts forward preliminary research hypotheses concerning this type of forearm lesion that is highly prone to missed diagnosis.

## 2 Patient information

This human subject case report was reviewed and approved by the Ethics Committee of Chuxiong Yi Autonomous Prefecture Hospital of Traditional Chinese Medicine (Approval No. 20260520002). Written informed consent was obtained from the patient's legal guardian for all diagnosis, treatment, radiographic imaging and clinical photography publication prior to manuscript submission, fully complying with the Declaration of Helsinki.

### 2.1 Chronological timeline of fractures and radiographic quantitative measurement

The patient was assigned the anonymized identifier S.U., a female child born on August 4, 2017.The patient suffered an accidental fall at home on August 24, 2021, at the age of 4 years old, presenting with pain and limited motion of the right forearm, and sought medical care at a local hospital. x-ray examination revealed a fracture of the middle and distal right radius with satisfactory alignment of fracture fragments. Conservative treatment including plaster immobilization and triangular bandage suspension of the right upper limb was administered. Follow-up radiography taken on September 1, 2021 showed dorsal angulation of the fractured radial segments, and transfer to a higher-level hospital was recommended (external hospital x-ray films were lost). The patient was admitted to our department on September 2, 2021. Physical examination on admission revealed pain and restricted range of motion of the right forearm. Closed manipulative reduction combined with small splint external fixation was performed for the middle-distal radial fracture.

**Figure 1:**
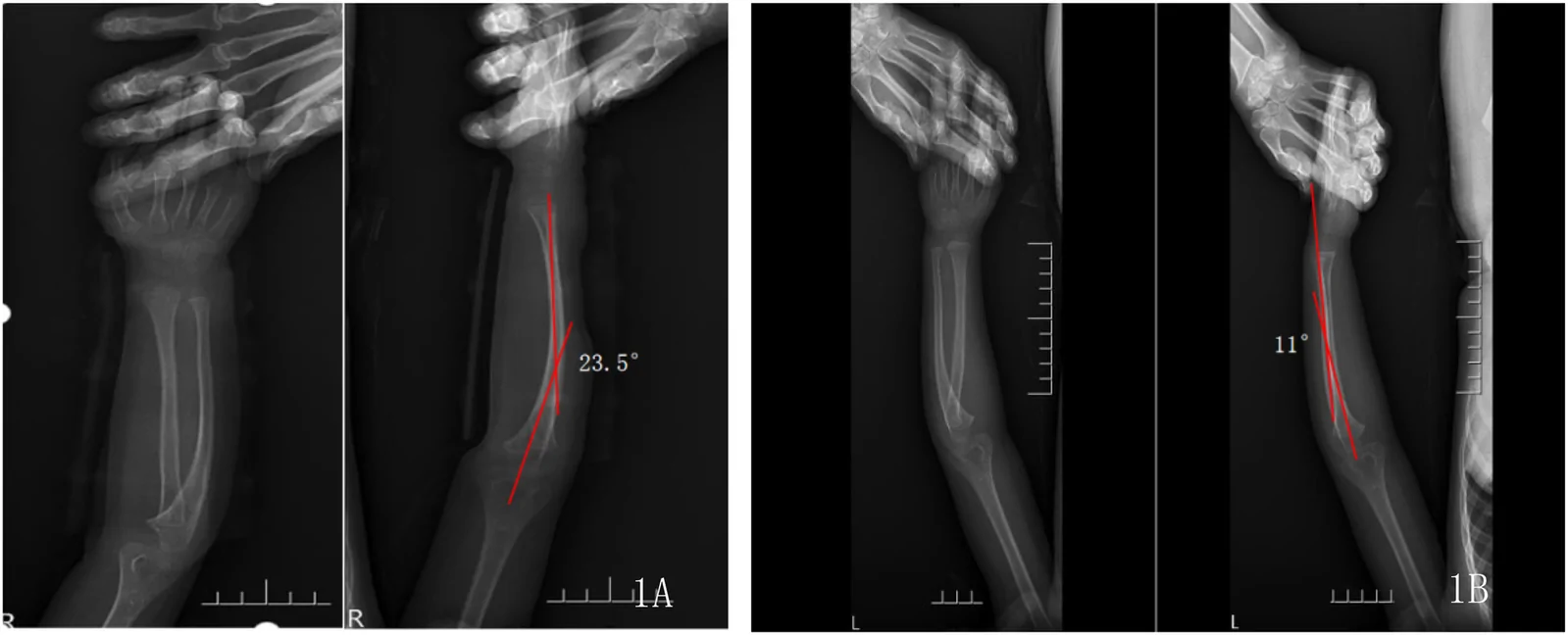
Imaging findings of the affected forearm after initial closed reduction **(A)**; contralateral intact left forearm radiograph **(B)**.

Radiographs after closed manipulative reduction revealed satisfactory reduction of the radial fracture, accompanied by sagittal bowing deformity of the right ulnar diaphysis measuring 23.5° (Figure 1A). The sagittal bow angle of the intact contralateral left ulna was merely 11°, and bilateral radiographic comparison confirmed the diagnosis of ulnar bowing fracture (Figure 1B). All angular measurements were performed on calibrated hospital Picture Archiving and Communication System (PACS) using the angulation method on lateral forearm radiographs: the central axes of the proximal and distal ulnar diaphyses were drawn and extended to intersect, and the sagittal included angle formed by the two lines was recorded as the ulnar bow angle.

**Figure 2:**
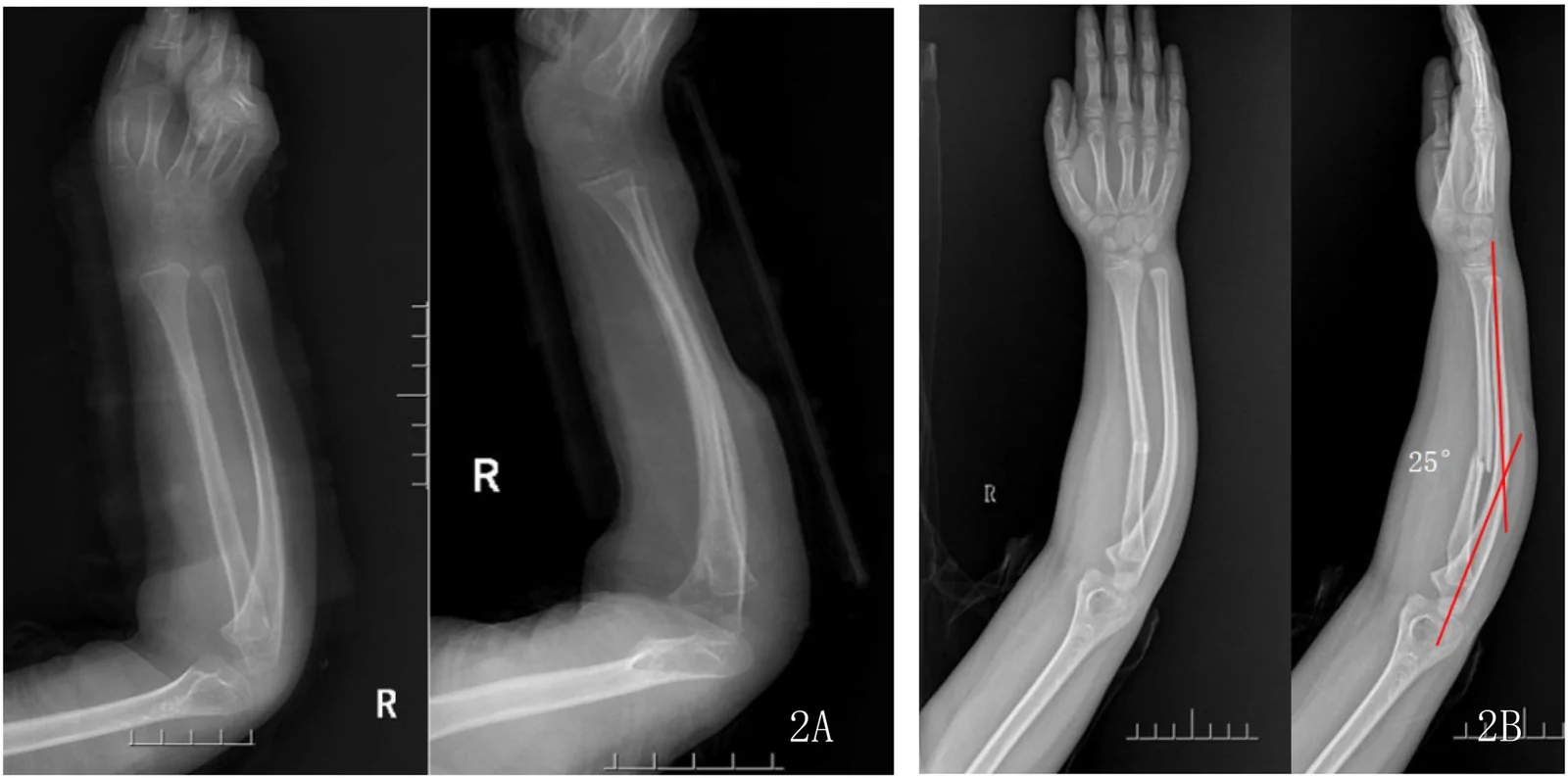
Four-week follow-up radiograph after small splint external fixation for the initial radial fracture **(A)**; right forearm radiograph after recurrent fall trauma four years later, showing persistent ulnar bowing deformity **(B)**.

Repeat x-ray examination four weeks after immobilization showed abundant callus formation at the middle-distal radial fracture site, indicating sound fracture healing. However, no callus or plastic remodeling was observed at the bowing deformity of the right ulnar diaphysis (Figure 2A). The small splint was subsequently removed, and the patient was discharged with routine follow-up instructions. No targeted intervention was carried out for the ulnar bowing deformity at that time. The child experienced no forearm pain, limited movement or other discomfort in daily life during the subsequent 4 years, so the guardian did not bring the patient back to our hospital for scheduled routine follow-up visits. No serial radiographs were obtained until the recurrent fall injury occurred in November 2025.

**Figure 3:**
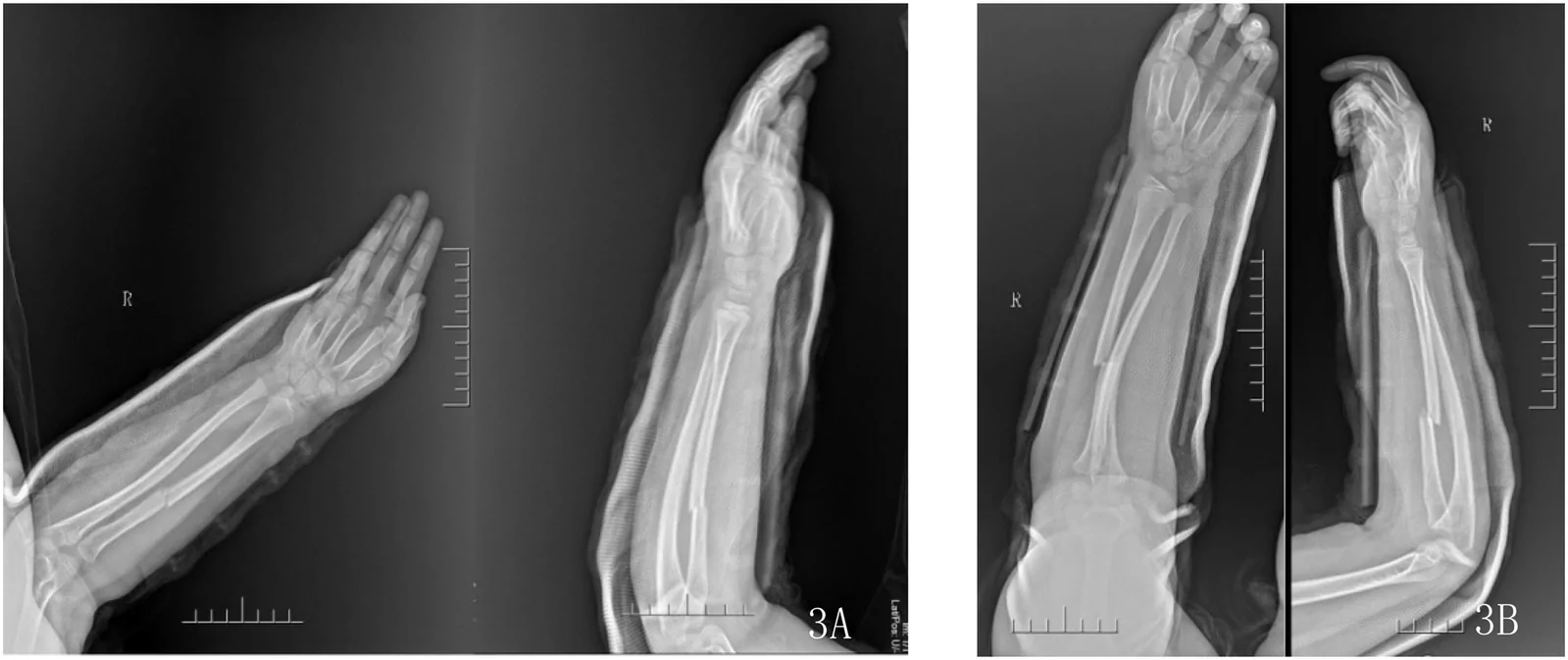
Post-manipulative reduction radiograph after refracture **(A)**; seven-day follow-up outpatient radiograph demonstrating secondary fracture displacement **(B)**.

On November 9, 2025 (age 8), the child fell at home and sustained an injury to the right forearm, presenting with immediate pain and limited mobility of the affected limb. The patient was rushed to our emergency department. Emergency radiographs demonstrated a fracture at the proximal-middle segment of the right radius with concomitant ulnar bowing deformity measuring 25°, and no spontaneous remodeling of the deformity was observed (Figure 2B). Manipulative reduction and plaster external fixation were performed in our outpatient department. Post-reduction radiography revealed acceptable fragment alignment (Figure 3A). A repeat outpatient follow-up visit was arranged on November 16, 2025, and follow-up x-ray demonstrated recurrent displacement of fracture fragments, verifying that conservative treatment failed to sustain satisfactory reduction (Figure 3B).

### 2.2 Operative details

**Figure 4:**
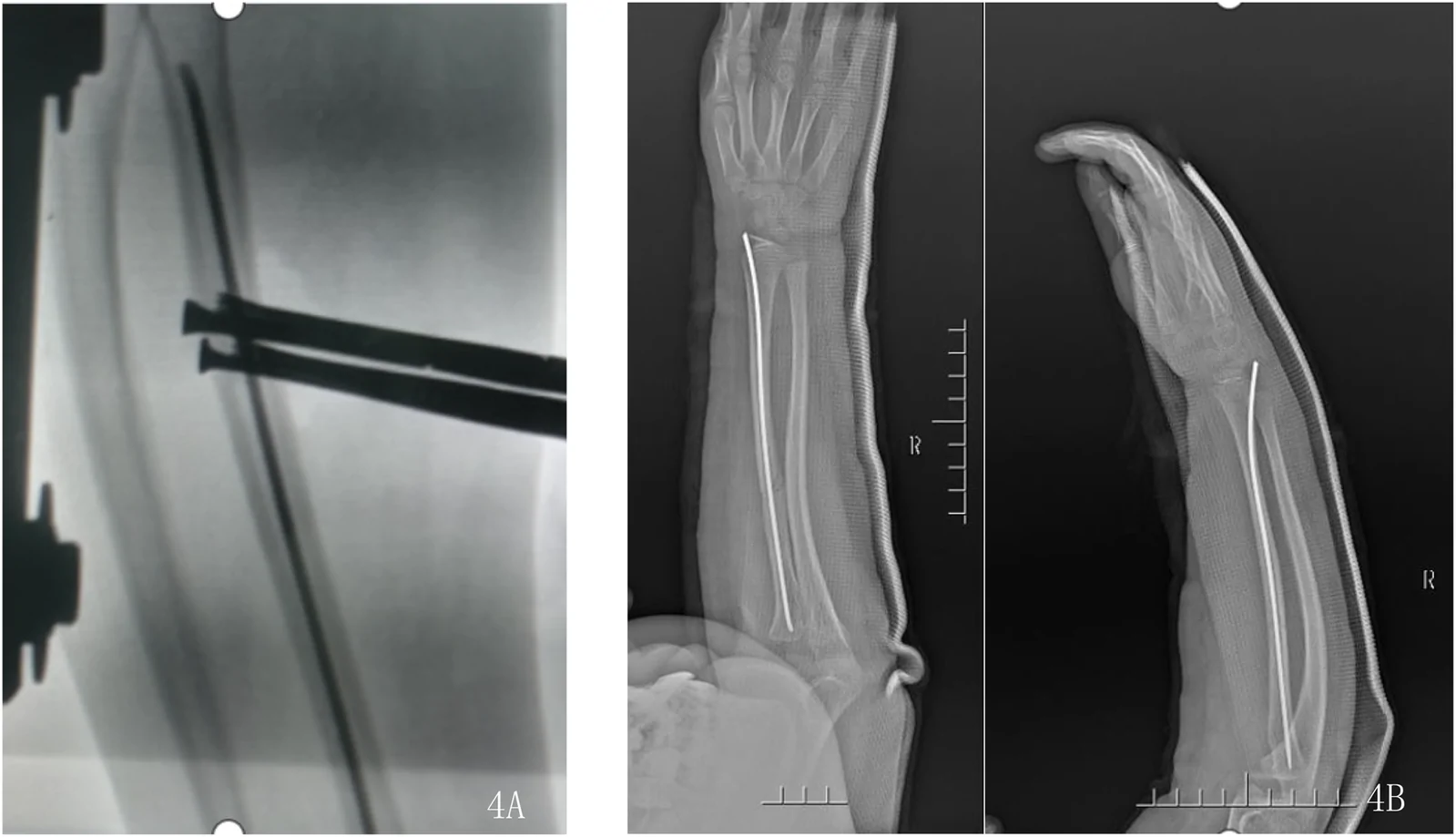
Intraoperative Fluoroscopic view showing anatomical reduction of radial fracture **(A)**; immediate postoperative radiograph confirming proper elastic intramedullary nail placement **(B)**.

Given the inability of conservative management to maintain fracture reduction and the potential risk of progressive forearm dysfunction secondary to unstable fracture, open reduction and elastic intramedullary nailing were carried out for the right radial fracture under general anesthesia. Under general anesthesia, open reduction and elastic intramedullary nailing were performed for the right radial fracture via a dorsal distal radial approach. The entry point was positioned 1.5 cm proximal to the physis at the metaphysis, directly posterior to Lister's tubercle, through which one 2.0-mm titanium elastic intramedullary nail was inserted. Open reduction was selected as the chronic ulnar bowing deformity disrupted the forearm biomechanical balance and rendered closed reduction unfeasible. No neurovascular injuries were encountered intraoperatively. Intraoperative fluoroscopy confirmed anatomical reduction of the radial fracture (Figure 4A), and postoperative radiographs revealed satisfactory nail placement and perfect fracture alignment (Figure 4B). No corrective osteotomy was performed for the pre-existing ulnar bowing deformity during surgery.

### 2.3 Postoperative rehabilitation and follow-up

**Figure 5:**
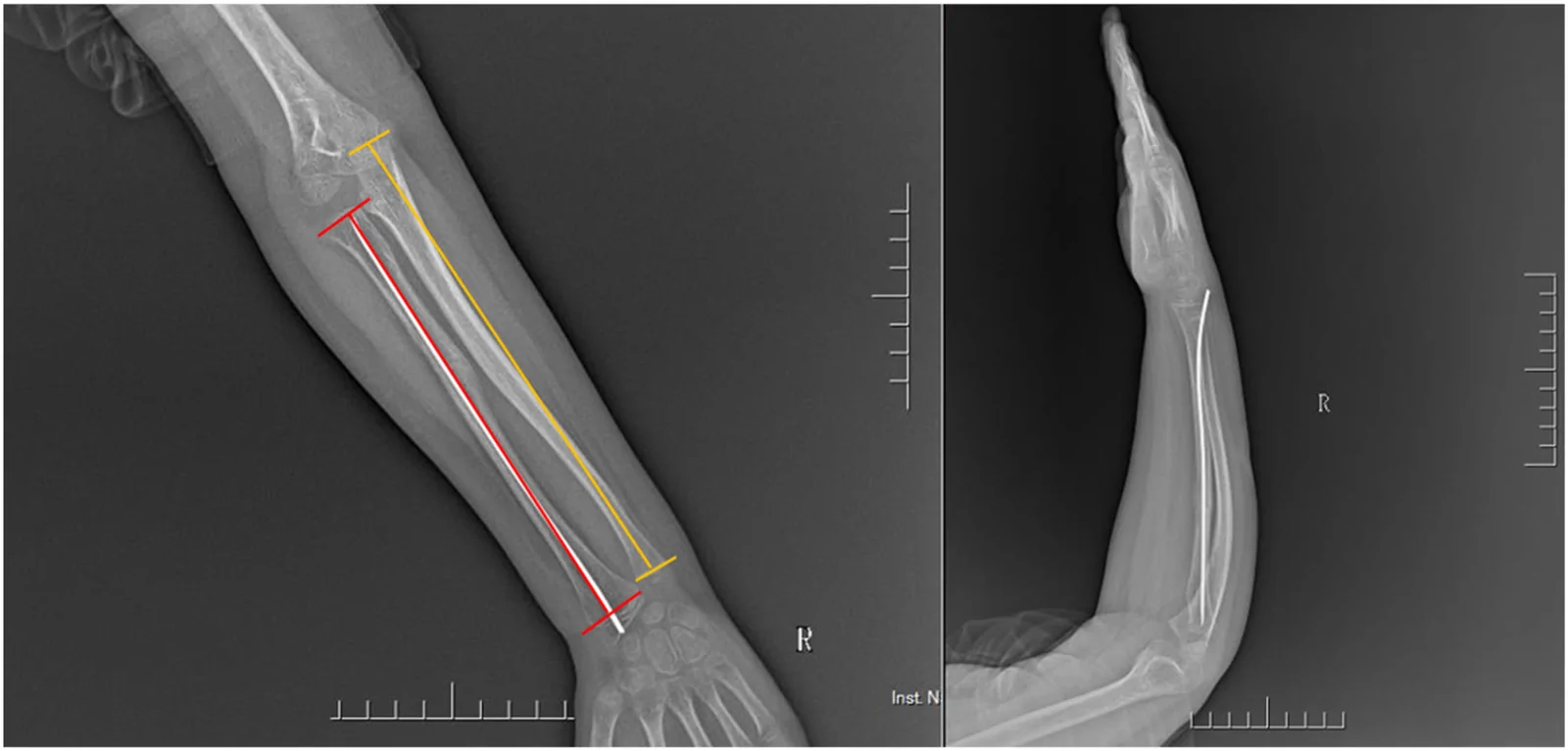
Two-month postoperative follow-up radiograph demonstrating solid radial fracture union; measured inner epiphyseal ulna-radius length ratio = 1.087.

Two-month postoperative radiographic follow-up: x-ray showed blurring of the radial fracture line and continuous callus formation at the fracture end, indicating good healing of the radial fracture. The elastic intramedullary nail was in a good position without loosening or displacement. The angulation of the old ulnar bowing deformity showed no significant change compared with the preoperative period, and no new bone injury or deformity progression was found (Figure 5).

**Figure 6:**
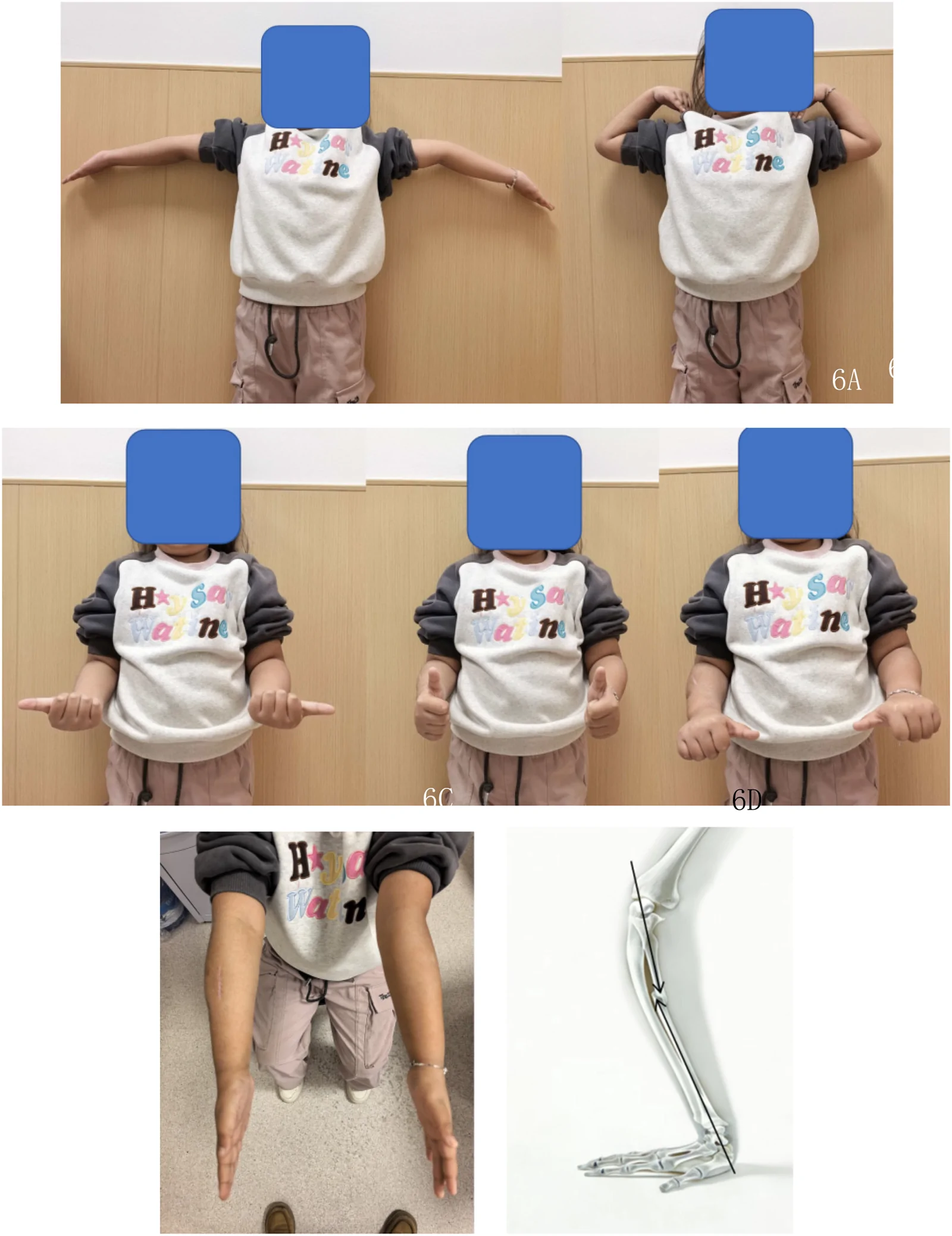
Postoperative two-month functional assessment: elbow flexion and extension range of motion **(A)**; forearm supination and pronation rotation function **(B)**; clinical appearance of the affected forearm with mild residual angular deformity **(C)**; schematic diagram showing abnormal stress distribution of forearm bone induced by chronic ulnar bowing deformity **(D)**.

Two-month postoperative functional follow-up revealed satisfactory recovery of elbow and forearm motion in the patient. Goniometric measurements were recorded as follows: the affected elbow achieved 142° of flexion and hyperextension of −5°, while the contralateral elbow presented hyperextension of −15°. Supination reached 88° and pronation reached 76° bilaterally (Figures 6A,B). Wrist flexion, extension, radial deviation and ulnar deviation were all within normal ranges. Pediatric Outcomes Data Collection Instrument (PODCI) assessment yielded a total upper-extremity score of 91 and a pain subscore of 96. The QuickDASH score was 6.8, with no wrist pain during routine grasping and writing activities. Only mild forearm angular deformity was observed on limb appearance (Figure 6C). The child's guardian fully accepted the residual mild cosmetic deformity, and the patient reported no pain or functional limitations in daily life. Long-term follow-up was scheduled to monitor forearm skeletal development and the stability of internal implants.

## 3 Discussion

### 3.1 Biomechanical causality between persistent ulnar bowing and radial refracture

Forearm bowing fracture in children refers to permanent plastic bending of the radial and/or ulnar diaphysis following trauma, and is classified as an incomplete fracture with the core characteristic of no definite cortical fracture line (5). If diaphyseal ulnar bowing remains uncorrected, relative ulnar shortening will alter interosseous membrane tension and the axial load transmission pathway of the forearm, leading to persistent stress concentration at the middle and proximal radial diaphysis — the anatomical site of refracture in this patient (Figure 6D). Finite element biomechanical simulations have verified that ulnar bowing induces abnormal tension of the forearm interosseous membrane, redistributes mechanical loads on the radial diaphysis, creates regional stress concentration, and ultimately triggers refracture (6). A prospective multicenter study reported a refracture rate of 6.7% among children with greenstick radial fractures accompanied by ulnar bowing (7). The patient in this study sustained a completely displaced radial refracture following a trivial fall, whereas the uninjured contralateral forearm endured equivalent physical activity loads over four years without fracture. This confirms that abnormal stress derived from ulnar bowing, rather than incidental trauma alone, acts as the core predisposing factor for refracture. Children's bone remodeling capacity declines with age; serial radiographs obtained over four years demonstrated spontaneous correction of the ulnar bow was absent. This case provides rare complete sequential imaging data documenting this pathological process across a 4-year follow-up period.

### 3.2 Effects of chronic ulnar bowing deformity on prognosis of forearm fractures in children and its radiographic assessment

As the stabilizing structural component of the forearm, intact ulnar morphology is essential for maintaining normal forearm biomechanical properties. Ulnar bowing directly disrupts forearm mechanical axes and ulnar length, resulting in a series of functional impairments. Combined bowing and shortening of the ulna disrupts the physiological length matching of the radius and ulna, predisposing to radial head dislocation or subluxation (8). Ulnar bowing also modulates interosseous space tension, and its mechanism of impact on forearm rotation differs from that of radial bowing; the two deformities often interact synergistically to produce complex combined malalignment. Radioulnar and ulnar bowing shifts the ulnar styloid and compromises ulnar-sided wrist stability, while volar or dorsal angulation directly causes incongruity of the distal radioulnar joint (DRUJ) (9). If chronic ulnar bowing is left uncorrected, diminishing skeletal remodeling potential with age results in permanent deformity. Patients with mild bowing and no obvious functional impairment may be managed conservatively. In the present case with chronic ulnar bowing, only the new radial fracture underwent reduction and fixation, yielding satisfactory postoperative forearm function. This indicates that deliberate correction of chronic ulnar bowing is unnecessary in such cases, and targeted repair of radial injury alone achieves favorable functional outcomes. Traditional assessment relying solely on diaphyseal angulation measurement is insufficient for evaluating forearm deformities. A comprehensive radiographic evaluation system incorporates three quantitative indicators: first, quantification of ulnar deformity severity via angulation measurement; second, the intraepiphyseal ulna-to-radius length ratio (IEUL/IERL) proposed by multicenter Monteggia fracture research, with a normal reference range of 1.047–1.141. This parameter reflects physiological radioulnar length matching and predicts the risk of radial head instability (10). The patient in this case exhibited 25° of ulnar angulation but an IEUL/IERL ratio of 1.087 within normal limits. No late radial head subluxation occurred during follow-up, and elbow function remained intact, suggesting routine measurement of this ratio to screen for joint instability in all children with ulnar angulation. Third, measurement of radial distal articular surface angles (radial inclination and volar tilt). Diaphyseal malalignment restricts forearm rotation, while morphological abnormalities of the distal radial articular surface disrupt DRUJ congruence, alter tension distribution within the triangular fibrocartilage complex (TFCC), cause ulnar-sided wrist impingement, and further limit pronation and supination (9, 11). Anatomical reduction of the radius in this patient restored physiological distal radial articular angles, eliminated abnormal wrist biomechanical loading, and fully recovered forearm rotational function. Therefore, integrated assessment combining diaphyseal angulation parameters and distal radial articular surface indices is required for complete evaluation of forearm deformity.

### 3.3 Treatment strategies for radial refracture combined with ulnar bowing

Individualized surgical planning is determined by age, deformity magnitude, forearm structural balance indices (IEUL/IERL ratio), and joint function (10, 12). Sanders et al. recommended conservative observation for children younger than 4 years with angulation deformity <20°, whereas closed reduction may be indicated for patients older than 6 years with deformity >10° (12, 13). However, current clinical guidelines lack standardized therapeutic recommendations for patients with recurrent forearm fractures complicated by chronic ulnar bowing. The patient in this case presented with isolated 25° ulnar bowing, a normal IEUL/IERL ratio, and intact elbow and wrist function; ulnar osteotomy for deformity correction was therefore unnecessary. Open reduction combined with elastic stable intramedullary nailing (ESIN) of the radius achieved satisfactory anatomical alignment and functional recovery without additional surgical trauma. Concurrent ulnar lengthening or corrective osteotomy is indicated for patients with severe ulnar bowing (>25°), abnormal radioulnar length ratios, or progressive deterioration of joint function to restore forearm mechanical alignment (10). Elastic intramedullary nailing represents an ideal minimally invasive internal fixation modality for recurrent long bone fractures in children after failed conservative management, as it protects the physes and permits early functional rehabilitation. Closed reduction and fixation failed in this patient due to disrupted forearm biomechanical balance secondary to chronic ulnar bowing, mandating open reduction. Intraoperatively, chronic ulnar malalignment only slightly hindered exposure of the interosseous membrane, which was easily resolved via gentle soft tissue retraction without additional complications.

### 3.4 Clinical implications and novel insights from this four-year follow-up case

This single long-term follow-up case yields three critical clinical recommendations for pediatric orthopedic practice:

1. Heightened diagnostic vigilance: Bilateral comparative full-length anteroposterior and lateral radiographs of the radius and ulna must be obtained for all children presenting with forearm trauma to screen for occult plastic ulnar bowing without clear fracture lines, a lesion frequently missed on initial presentation. Fixation of radial fractures without addressing concurrent plastic ulnar deformity carries long-term refracture risk.
2. Reference for therapeutic decision-making: School-age children with mild isolated ulnar bowing, balanced radioulnar epiphyseal length ratios, and complete upper-extremity function may undergo radial fixation with elastic intramedullary nails alone, avoiding the additional trauma of ulnar osteotomy. Concurrent corrective osteotomy is required for severe deformity combined with joint instability.
3. Value for research hypothesis generation: This long-term serial follow-up case provides authentic clinical observational data for subsequent large-cohort studies investigating the critical threshold of ulnar bowing angle associated with elevated radial refracture risk, and offers a representative clinical phenotype for finite element biomechanical modeling of the forearm. As a single-case analysis, the conclusions drawn cannot be generalized to all comparable patients and only serve as preliminary hypotheses for future prospective validation research.

### 3.5 Study limitations

This report is limited to a single pediatric patient, with short-term functional follow-up restricted to 2 months. Extended monitoring of skeletal growth is necessary to observe long-term morphological changes of the forearm. Large-scale multicenter prospective trials are required to validate the stratified treatment hypothesis proposed herein.

## 4 Conclusions

Forearm bowing fractures in children feature an insidious onset and complex management workflow. Early accurate diagnosis combined with multi-index quantitative radiographic assessment (ulnar bowing angle, IEUL/IERL ratio, distal radial articular surface angles) is the cornerstone of preventing residual deformity and recurrent injury. For children with displaced recurrent radial fractures complicated by mild chronic ulnar bowing, balanced radioulnar epiphyseal length ratios, and intact elbow and wrist function, open reduction of the radius combined with elastic intramedullary nailing alone effectively restores radial anatomical alignment and achieves favorable short-term limb function. Ulnar corrective osteotomy is not required for this specific patient subgroup. Observations from this single case only establish preliminary stratified treatment hypotheses and lack sufficient evidence for universal clinical application; the proposed protocol must be validated by large prospective cohort trials prior to widespread clinical adoption.

## Data Availability

The datasets presented in this article are not readily available because of ethical and privacy restrictions. Requests to access the datasets should be directed to the corresponding author.
